# Cite what you read, read what you cite

**DOI:** 10.1016/j.patter.2025.101344

**Published:** 2025-08-08

**Authors:** Andrew L. Hufton

**Affiliations:** Editor-in-chief, *Patterns*, Cell Press

## Main text

You read a preprint that inspired your research. Later, when you are ready to publish your own findings, you notice that the final peer-reviewed version has been published in a journal. Which version should you cite?

Preprints are early versions of papers shared online prior to peer-review and formal publication. The term is now somewhat anachronistic since many journals are no longer printed, but the name has stuck and the concept has gained traction in many fields. *Patterns* strongly supports preprint sharing and believes that preprints play a crucial role in promoting open science. The presence of preprint versions, however, can sometimes create confusion about the right way to cite a work.

An author may prefer to cite the final peer-reviewed version due to journal policies or simply out of courtesy to the authors. This is well-intentioned, and indeed Cell Press policies encourage the citation of the final peer-reviewed version over the preprint. This can, however, create a situation where an author is citing a version of a paper that they have not read. Claims and conclusions can change during the peer-review process. In the extreme, this can lead to authors inadvertently misrepresenting the conclusions in a paper. A number of studies have observed that findings are frequently cited inaccurately (see, e.g., Todd et al., 2010, *Mar. Ecol. Prog. Ser.* or Greenberg, 2009, *BMJ*). The reasons for these errors are diverse, but one contributing issue may simply be that researchers aren’t always reading the same version they are citing.

It is therefore crucial that authors accurately cite what they have actually read. Whenever possible, authors should make a good faith attempt to obtain and read the final peer-reviewed version of a paper before they cite it. In some cases, we will allow authors to cite both the final peer-reviewed version and the preprint version if that allows them to provide a more honest accounting of their sources or if they simply want to highlight an open-access version of a key paper. When quoting from a paper directly, authors should take special care to ensure that their quote matches exactly with the content in the version they have cited.

In general, if a paper is cited, a reader should be able to assume that the citing authors have read it. Whenever this is not the case, this should be made clear in the text of the paper. If, for example, natural language processing methods or AI models were used to extract information from papers, these methods should be transparently and reproducibly described in the paper. Similarly, if an author wishes to cite a paper they haven’t read because it was cited by another source they did read, this should be made clear to readers. This may be necessary in cases where the primary source is no longer accessible (for a cautionary look, however, into how indirect citation can lead to the propagation of scientific myths, see Rekdal, 2014, *Soc. Stud. Sci.*).

Similar issues can sometimes emerge when citing data, code, or models. *Patterns* supports formal citation of data, code, or model records when they are archived at formal scholarly repositories and the repository provides clear citation information. Authors may also choose to cite a related peer-reviewed publication to help give credit to the creators. The data, code, or model creators may even ask for a specific paper to be cited. Again, as with preprints, it is always best practice to read a paper before citing it. Be polite but be rigorous.

Ultimately, we need to respect the original function of citations—providing a robust and scholarly record of the sources that underlie and support a work. Cite what you read or used honestly and accurately.

